# Energy Harvesting Using Thermocouple and Compressed Air

**DOI:** 10.3390/s21186031

**Published:** 2021-09-09

**Authors:** Robert Bayer, Jiří Maxa, Pavla Šabacká

**Affiliations:** 1 Department of Electrical and Electronic Technology, Brno University of Technology, 61100 Brno, Czech Republic; xbayer02@vutbr.cz (R.B.); xhlava44@vutbr.cz (P.Š.); 2 Institute of Scientific Instruments of the CAS, Královopolská 147, 61264 Brno, Czech Republic

**Keywords:** Peltier–Seebeck effect, Laval nozzle, harvester thermocouple, conical shockwave, perpendicular/detached shockwave, energy harvesting

## Abstract

In this paper, we describe the possibility of using the energy of a compressed air flow, where cryogenic temperatures are achieved within the flow behind the nozzle, when reaching a critical flow in order to maximize the energy gained. Compared to the energy of compressed air, the energy obtained thermoelectrically is negligible, but not zero. We are therefore primarily aiming to maximize the use of available energy sources. Behind the aperture separating regions with a pressure difference of several atmospheres, a supersonic flow with a large temperature drop develops. Based on the Seebeck effect, a thermocouple is placed in these low temperatures to create a thermoelectric voltage. This paper contains a mathematical-physical analysis for proper nozzle design, controlled gas expansion and ideal placement of a thermocouple within the flow for best utilization of the low temperature before a shockwave formation. If the gas flow passes through a perpendicular shockwave, the velocity drops sharply and the gas pressure rises, thereby increasing the temperature. In contrast, with a conical shockwave, such dramatic changes do not occur and the cooling effect is not impaired. This article also contains analyses for proper forming of the head shape of the thermocouple to avoid the formation of a detached shockwave, which causes temperature stagnation resulting in lower thermocouple cooling efficiency.

## 1. Introduction

**Figure 1:**
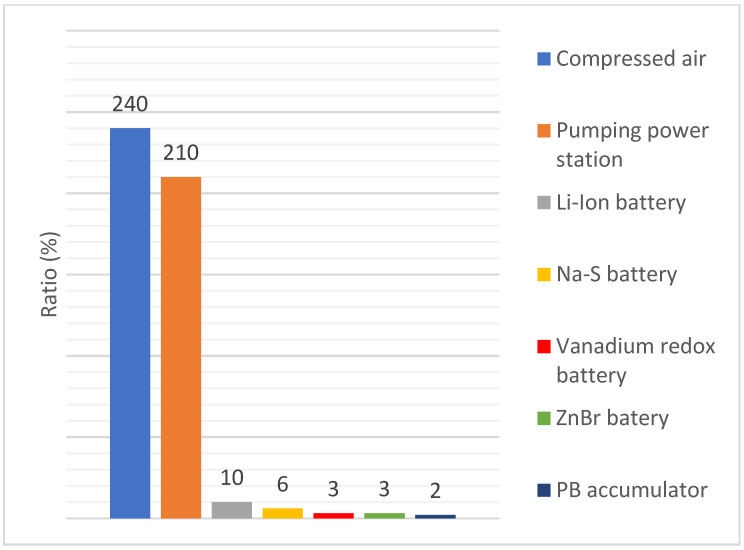
Ratios of energy stored on energy invested into construction of an energy storage.

Just like batteries, compressed air is a secondary energy source. The study of CH. J. Barnhart proves that compressed air is the most efficient regarding energy stored on invested (ESOI) value, which shows the ratio between the amount of energy stored in a secondary energy source and the amount of energy required for its construction [1]. Figure 1 shows the comparison of values of an ESOI of some secondary energy sources.

Gases warm up when compressed and cool when they expand, and these two phenomena are not commonly utilized in practice. As a part of research in the field of vacuum pumping of chambers in an environmental scanning electron microscope (ESEM) through apertures, another study is taking place at Department of Electrical and Electronic Technology at Brno University of Technology with the help of the Institute of Scientific Instruments of the Czech Academy of Science. It is focused on utilizing the rapid temperature drop within a supersonic air flow behind a nozzle to create a thermoelectric voltage using the Seebeck effect.

## 2. Materials and Methods

To utilize the Seebeck effect, the cold side of a set of thermocouples was placed into the flow to greatly cool them, and the warm side of the thermocouples was put into a gas reservoir containing a pressurized gas at room temperature. On the contrary, when filling and pressurizing the reservoir with a gas using a closed nozzle in the chamber, the warm side of the thermocouple was warmed up by the incoming gas and the cold side behind a closed aperture was at room temperature.

### Experimental Chamber

**Figure 2:**
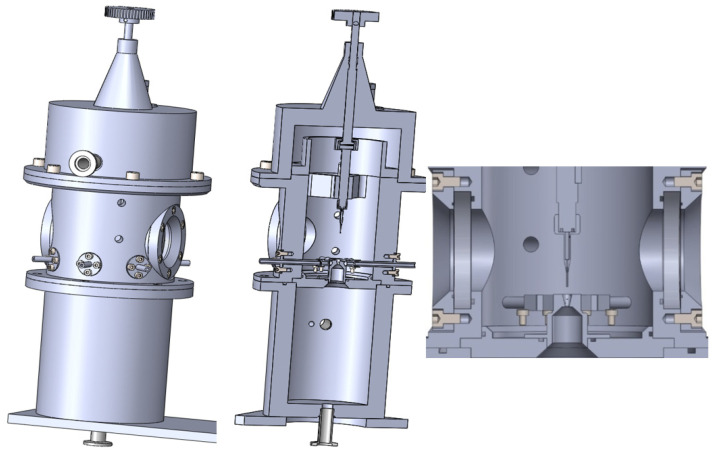
Experimental chamber.

The experimental chamber consisted of two chambers separated with a small aperture, simulating the differential pumping of gas within an ESEM. The experimental chamber also contained an interchangeable part separating the two inner chambers, allowing us to change the size and shape of the aperture, and a sliding holder for mounting different sensors and devices, as shown on Figure 2.

To research the possibility of using the Seebeck effect, a theoretical analysis and mathematical-physical analysis were necessary to find the right aperture dimensions and electrode shape.

**Figure 3:**
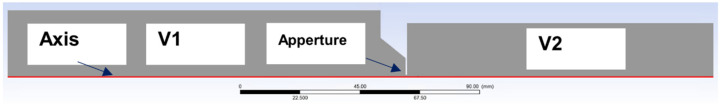
Two-dimensional axisymmetric model.

Figure 3 shows the layout of inner chambers V1 and V2 separated with a nozzle as used for calculations in the Ansys Fluent system. The whole simulation was calculated as a 2D axisymmetric. Air was used in the analyses.

Mathematical-physical analyses in the Ansys Fluent system, where the pressure-based solver setting with second-order discretization was used, showed the following pressure and temperature waveforms as a function of time. These calculations were performed as 2D axisymmetric time variable calculations.

Boundary conditions are described in Figure 3. The mesh was formed from hexagon elements of 0.5 mm in size in rectangular sections. The narrowing region with aperture was created from triangular elements with a gradual refinement, a growth rate of 1.05 to an element size of 0.01 mm in the aperture region, and the region of supersonic flow behind the aperture. Monitor check convergence absolute criteria were set to 0.001 for continuity and velocity. The value for energy was set to 1 × 10^−6^.

In the first experiment, supersonic flow with velocity *M_v_* = 2.6 was used.

The experimental chamber construction comes from the physical theory of isentropic one-dimensional flow.

Relationships set the ratios of pressures, densities, velocity, and Mach number between the area of nozzle input, in the nozzle and within the computational cross-section of the gas expansion behind the nozzle [2].

For isentropic flow, the following relationships apply:

(1) $$\frac{v_{v}}{v_{kr}}={\left[\frac{\left(\varkappa +1\right)M^{2}}{2+\left(\varkappa -1\right)M^{2}}\right]}^{\frac{1}{2}},$$

(2) $$\frac{v_{v}}{v_{o}}={\left[\frac{2}{2+\left(\varkappa -1\right)M^{2}}\right]}^{\frac{1}{2}},$$

(3) $$\frac{T_{v}}{T_{o}}=\frac{2}{2+\left(\varkappa -1\right)M^{2}},$$

(4) $$\frac{p_{v}}{p_{o}}={\left[\frac{2}{2+\left(\varkappa -1\right)M^{2}}\right]}^{\frac{\varkappa }{\varkappa -1}},$$

(5) $$\frac{\rho_{v}}{\rho_{o}}={\left[\frac{2}{2+\left(\varkappa -1\right)M^{2}}\right]}^{\frac{1}{\varkappa -1}},$$

(6) $$\frac{\rho_{v}}{\rho_{kr}}=\frac{A_{kr}}{A}=M{\left[\frac{\varkappa +1}{2+\left(\varkappa -1\right)M^{2}}\right]}^{\frac{1\varkappa +1}{2\varkappa -1}},$$

where *p_o_* is the input pressure, *p_v_* is the output pressure, *T_o_* is the input temperature, *T_v_* is the output temperature, *v_o_* is the input velocity, *v_v_* is the output velocity, *v_kr_* is the critical velocity, *ρ_o_* is the input density, *ρ_v_* is the output density, *M* is the Mach number, ϰ is the gas constant = 1.4, *A* is the computational cross-section and *A_kr_* is the critical cross-section.

In order to achieve a supersonic flow with velocity *M_v_* = 2.6 behind the nozzle, a ratio between *p_v_* and *p_o_* needs to be 1:4 according to Equation (4). Considering the output pressure behind the nozzle will be 1 atm, the chamber before the nozzle should be pressurized to 4 atm or more precisely 371,971 Pa [3].

The designed nozzle diameter was 1.5 mm, with 0.5 mm length and opening angle 12° according to Daněk [4]. The next step was to calculate the appropriate shape and spot to place the probe with the thermocouple system [5,6], which is crucial to maximize the utilization of the cryogenic temperature within the supersonic flow [7,8,9,10].

This cryogenic temperature is based on the energy equation:

(7) $$e=\frac{v^{2}}{2}+c_{p}T,$$

where *e* is energy, *v* is flow velocity, *c_p_* is heat capacity at constant pressure, *T* is temperature of the gas flow.

**Figure 4:**
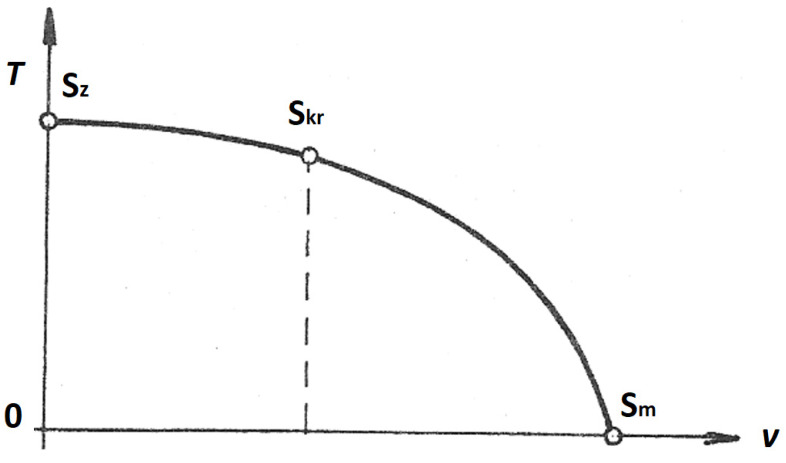
The temperature parabola.

The equation gives the so-called temperature parabola (Figure 4), which determines the dependence in which the temperature of the gas decreases with accelerating flow.

In the temperature parabola, *S_z_* denotes the decelerated state where the velocity is zero, *S_kr_* is the critical state where the velocity reaches *V_kr_* at the narrowest point in the nozzle, and *S_m_* denotes the limit state where the velocity reaches a theoretical maximum that cannot occur in practice. The temperature at that point would reach 0 K.

In our case, with the probe placed as described, we were operating in the region between *S_kr_* and *S_m_*.

## 3. Results

**Figure 5:**
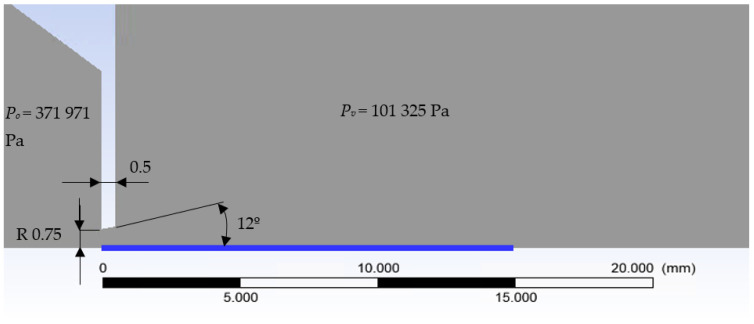
Two-dimensional axisymmetric model.

**Figure 6:**
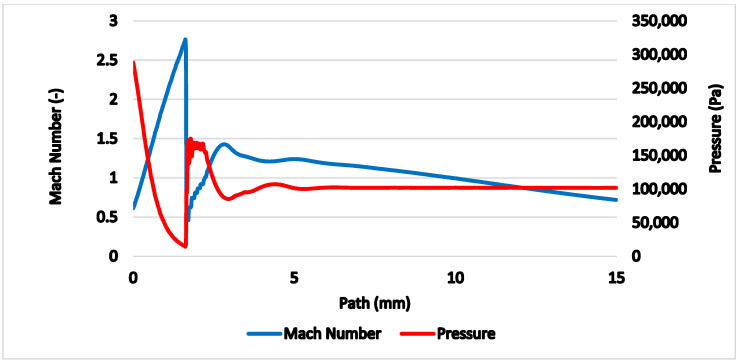
Mach number and pressure on the axis of the flow.

**Figure 7:**
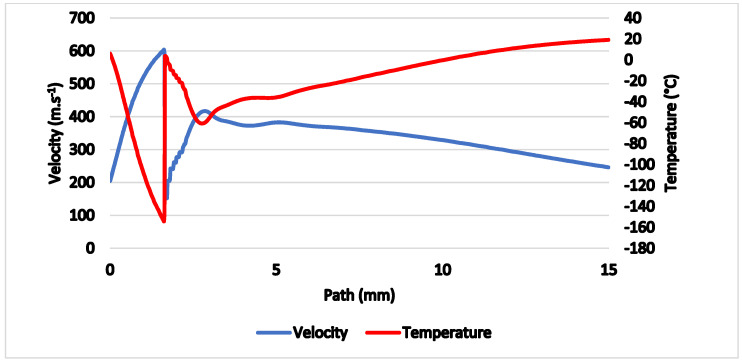
Velocity and temperature on the axis of the flow.

**Figure 8:**
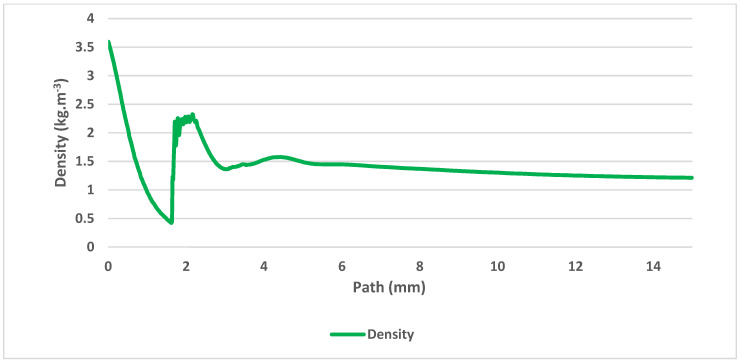
Density on the axis of the flow.

The gas flow running through the nozzle described above is a typical example of a critical flow [11,12]. Figure 5 shows the path behind the nozzle without the probe inserted, on which the course of pressure, Mach number (Figure 6), temperature, velocity (Figure 7) and density (Figure 8) are calculated.

When considering the calculated pressure ratio between *p_o_* = 371,971 Pa and *p_v_* = 101,325 Pa, the relationships 1 to 6 mentioned above give us following results as shown in Table 1:

The speed of sound on the input in the given environment is *v_o_* = 347.2 m·s^−1^ and is determined from relationship 8:

(8) $$v_{o}=\sqrt{\chi R_{A}T_{o}},$$

where *R_A_ =* 287.039 J·kg^−1^K^−1^ is the gas constant for air and *T_o_* = 300 K [13], ϰ is the Poisson’s ratio = 1.4.

In Table 1 the ratio of *v_v_*/*v_o_* = 0.6521, which gives the value of *v_v_* = 588.6 m·s^−1^ when using relationships mentioned above.

Similarly, the gas density on the input *ρ_o_* = 4.32 kg·m^−3^ can be calculated from ideal gas state Equation (9):

(9) $$\rho_{o}=\frac{p_{o}}{RT_{o}}.$$

In Table 1, the ratio *p_v_*/*p_o_* = 0.0617. Then it is possible to determine the value of output density *ρ_v_* = 0.51 kg·m^−3^.

Similarly, the ratio of temperatures in the input and output where *T_o_* = 300 K allows calculation of the temperature in the output *T_v_* = 127.56 K.

We made a back check after the mathematical–physical analyses in the Ansys Fluent system according to the one-dimensional flow physics for the computational cross-section. We compared the theoretically obtained values with the values obtained using Ansys Fluent and the measurement errors were minimal.

The values of *v_v_*, *ρ_o_*, *ρ_v_*, and *T_v_* were used as control values for results obtained using the Ansys Fluent system as shown in Table 2:

The results prove an exact match between the mathematical-physical analyses obtained with Ansys Fluent and the theory of physics of isentropic one-dimensional flow [14,15,16]. The supersonic flow ends with a characteristic shockwave at distance of 1.6 mm from the critical cross-section of the nozzle.

As a next step, an analysis of the location of the Mach disk according to the relationship 10 was performed [17]:

(10) $$z_{M}=0.67D_{kr}\sqrt{\frac{P_{o}}{P_{1}}}=1.93\;\mathrm{mm}.$$

**Figure 9:**
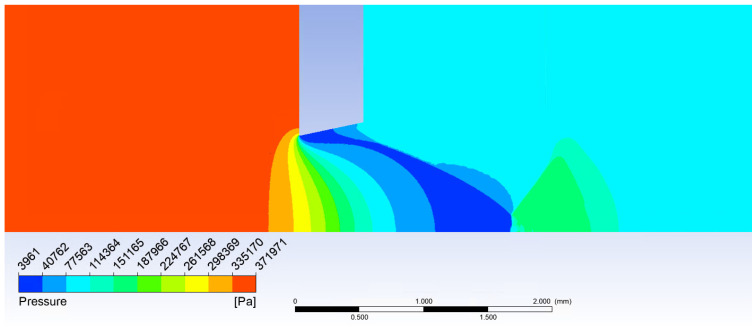
Mach disk calculated in Ansys Fluent.

Figure 9 proves the Mach disk from Ansys Fluent analysis is also located at the position assumed according to the theory.

**Figure 10:**
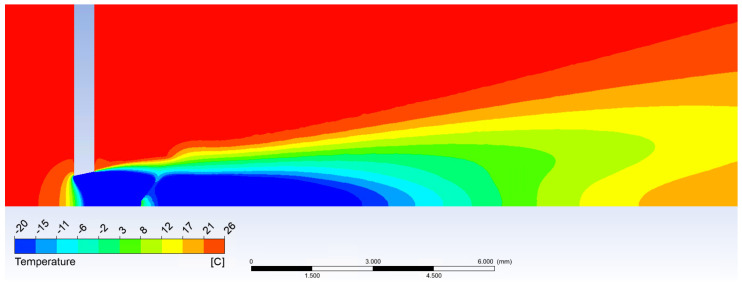
Temperature calculated in Ansys Fluent.

**Figure 11:**
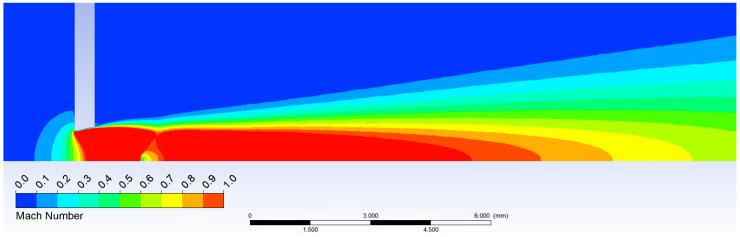
Mach number calculated in Ansys Fluent.

According to theoretical values and analyses gained from Ansys Fluent shown above and in Figure 10 and Figure 11, it is evident the probe should be placed before the shockwave at 1 mm from the critical cross-section to the tip of the probe.

Considering a supersonic flow (*Mv* > 1) a shockwave forms at a specific distance before any obstacle put into the flow. At first the gas is slowed down in a non-isentropic way to subsonic speed near the shockwave and then it slows down in the isentropic way to zero velocity at a stagnant point at the head of the obstacle. When the gas stagnates at the head of the probe, the temperature rises, lowering the effectiveness of cooling of the thermocouple. The probe should have an appropriate tip allowing formation of a conical shockwave, where state variables do not change that rapidly, instead of the perpendicular shockwave [18,19,20].

**Figure 12:**
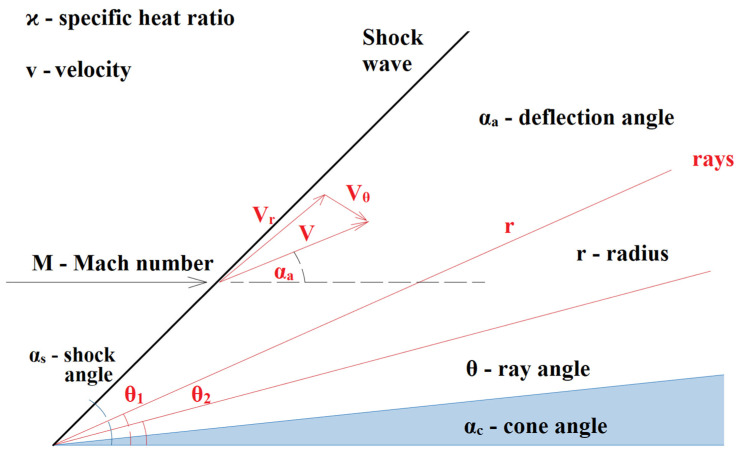
Taylor–McCall theory [21].

The relationship between the cone angle and the shock angle and thus the point of the detachment of the shockwave and its change from conical shockwave to perpendicular shockwave is described by the Taylor–McCall theory as shown in Figure 12.

The Taylor–McCall theory can be described with relationship 11:

(11) $$\frac{\varkappa -1}{2}\left[1-v_{r}^{2}-{\left(\frac{dv_{r}}{d\theta }\right)}^{2}\right]\left[2v_{r}+cot\theta \frac{dv_{r}}{d\theta }+\frac{d^{2}v_{r}}{d\theta^{2}}\right]-\frac{dv_{r}}{d\theta }\left[v_{r}\frac{dv_{r}}{d\theta }+\frac{dv_{r}}{d\theta }\frac{d^{2}v_{r}}{d\theta^{2}}\right]=0,$$

where *ϰ* is the specific heat ratio, *v* is velocity, *M* is the Mach number, *s* is shock angle, *a* is the deflection angle, *r* is the radius, *θ* is the ray angle and *c* is the cone angle.

**Figure 13:**
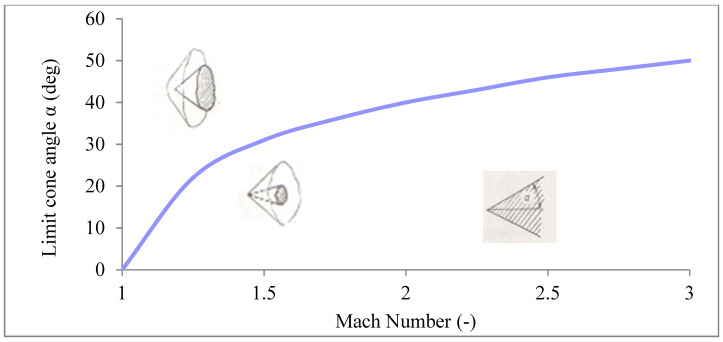
Dependence of Mach number on a probe cone angle [4].

This relationship can be seen in Figure 13, where the area above the curve represents conditions for perpendicular shockwave forming and the area below the curve represents conditions for conical shockwave forming.

**Figure 14:**
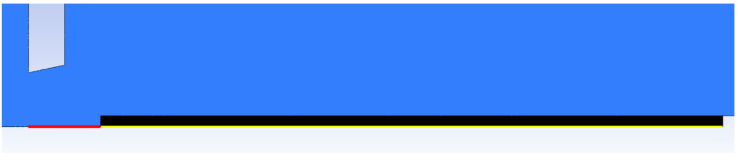
Probe with cylinder head.

**Figure 15:**
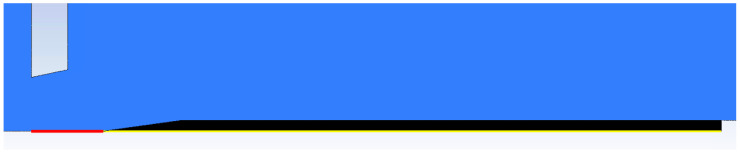
Probe with cone head.

For better demonstration of different behavior of the flow there are two variants of probe shapes shown. The first probe without the modified head that causes a perpendicular shockwave formation can be seen in Figure 14. The second probe with a cone-shaped tip with an 8° angle dimensioned to create a conical shockwave and to be suitable for use even in slower gas flow can be seen in Figure 15.

In Figure 14 and Figure 15, there are paths highlighted in red and yellow for which all calculations were performed. The red path is 1 mm long and leads from the critical cross-section of the nozzle to the tip of the probe. The yellow path is 8.6 mm long and runs along the axis of the probe.

**Figure 16:**
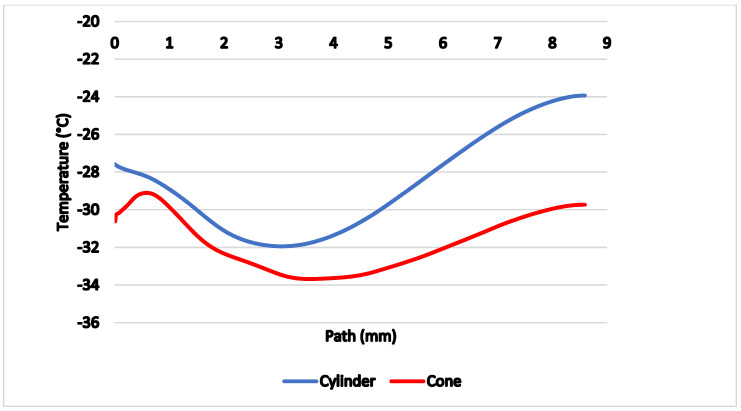
Comparison of temperatures along the probe axis.

Figure 16 shows comparison of temperatures along the axis of the probe with thermocouple (yellow path), proving the conical probe reached lower temperatures by 2 to 3 °C on average compared to the cylindrical probe.

The reason for the lower temperature on the yellow track is precisely the formation of a conical shockwave, beyond which there is no sharp drop in velocity and thus no high stagnation temperature, which would invalidate the desired effect. On the contrary, the temperature drops to the tip of the cone, whereas in a cylindrical probe it increases.

**Figure 17:**
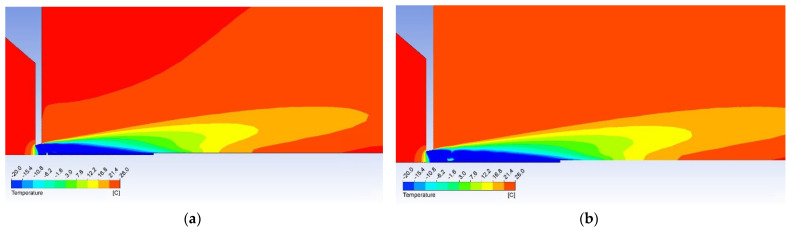
Temperature maps of gas flow with (**a**) the cylindrical probe and (**b**) the conical probe.

The temperature map can be seen in Figure 17 showing the higher temperature is evident before the head of the cylindrical probe and the lower temperature zone being shorter. This means the conical probe can be longer and more thermocouples can be installed within resulting in more power gained.

The reason for the higher temperature at the head of the cylindrical probe is the perpendicular shockwave formed before the cylindrical probe. At flow velocity above 50 m·s^−1^ the head of the cylindrical probe reaches the stagnation temperature *T_stg_*, which is higher than the static temperature *T* without any obstacle which we need for maximum cooling of the thermocouple probe. The following relationship applies:

(12) $$\frac{T}{T_{stg}}={\left(1+\frac{\varkappa -1}{2}M^{2}\right)}^{-1}.$$

**Figure 18:**
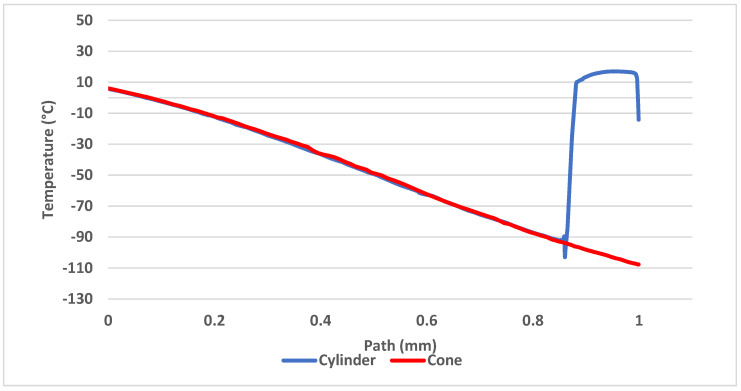
Temperature development between the nozzle and the head of both probe variants.

Previously shown results (Figure 6 and Figure 7) show that in 1 mm distance from the critical cross-section of the nozzle at the location of the head of the cylindrical probe the static temperature reaches *T* = −108 °C (165 K) and velocity reaches *M_v_* = 2. This means the stagnation temperature is *T_stg_* = 17 °C (290 K), which can be seen in Figure 18. The stagnation temperature is 17 °C because the following temperature drop is caused by the cooled probe behind the shockwave as seen in Figure 17a.

**Figure 19:**
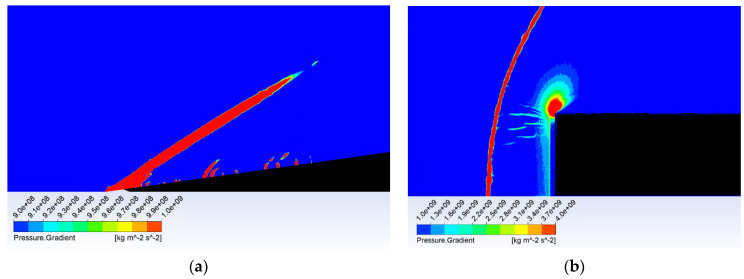
Shockwave forming before the probe head: (**a**) conical shockwave, and (**b**) perpendicular/detached shockwave.

Figure 19 shows the formation of (a) a conical shockwave, (b) a perpendicular/detached shockwave, visualized with pressure gradient.

**Figure 20:**
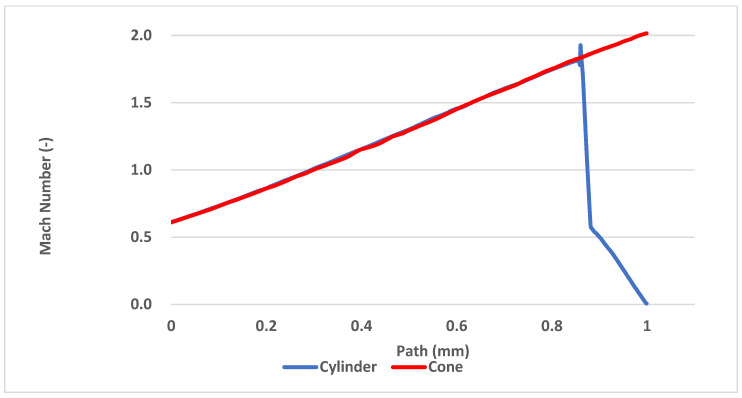
Mach number development between the nozzle and the head of both probe variants.

Another way to compare the results from the mathematical-physical analyses with the theoretical ones can be the comparison of the Mach number of both probe variants. According to the results, the conical probe does not affect the flow thanks to the conical shockwave and the cylindrical probe slows the flow down to the stagnation velocity, as shown in Figure 20.

**Figure 21:**
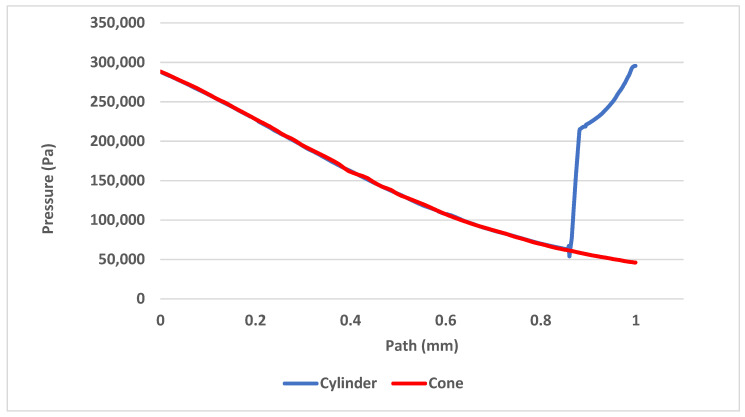
Pressure development between the nozzle and the head of both probe variants.

As a result of the flow slowing before the cylindrical probe, the pressure rises rapidly whereas no pressure change can be seen before the conical probe, as shown in Figure 21.

The temperature, Mach number and pressure values with the conical probe used (Figure 18, Figure 20 and Figure 21) match perfectly with the theoretical ones for the unaffected free gas flow (Figure 6 and Figure 7).

## 4. Conclusions

The article follows up on the research carried out at the Department of Electrical and Electronic Technology at Brno University of Technology in cooperation with the Institute of Scientific Instruments of the Czech Academy of Science, in the field of critical flow in nozzles in supersonic mode. The article describes mathematical-physical analyses to serve as a basis for the modification of the already existing experimental chamber for an experiment dealing with the use of critical flow of compressed air from the nozzle, when there is a sharp drop in temperature. It was necessary to perform analyses to obtain a suitable nozzle shape, allowing a controlled gas expansion and suitable placement of the probe in the flow, in order to use the potential of reduced temperature before the shockwave.

Analyses were also performed to properly shape the probe head to prevent the formation of a perpendicular shockwave that would cause a stagnation temperature and reduce the cooling efficiency of the probe.

The paper is a continuation of research carried out at the Department of Electrical and Electronic Technology of Brno University of Technology in cooperation with the Institute of Scientific Instruments of the Czech Academy of Sciences in the field of critical flow in jets in supersonic mode. The paper describes mathematical and physical analyses used as a basis for the modification of an existing experimental chamber for an experiment dealing with the use of critical flow of compressed air from a nozzle during a sharp drop in temperature. In order to exploit the potential of the reduced temperature prior to the shockwave, analyses were required to obtain a suitable nozzle shape to allow controlled gas expansion and appropriate placement of the probe in the flow.

Analyses were also performed to properly shape the probe head to prevent the formation of a perpendicular shockwave that would cause temperature stagnation reducing the cooling efficiency of the probe.

Mathematical and physical analyses were performed for a pressure gradient of 3.7:1 separated by a 1.5 mm diameter aperture producing a supersonic flow of up to 2.6 Mach with a significantly reduced temperature region down to −150 °C. Probes were placed in this flow and flow analysis was carried out around a cylindrical probe that produces a perpendicular detached shockwave in the supersonic flow and around a conical probe tip variant with calculated angle parameters such that it produced a conical shockwave. The results showed a significant negative effect of the perpendicular shockwave on the desired probe cooling results due to the fact that the perpendicular shockwave ended the supersonic flow and thus the state variables behind it changed dramatically. The cone shockwave does not have these properties and the temperature behind the cone shockwave remained low.

The given analyses are the basis for a forthcoming experiment in the experimental chamber. This experiment will be carried out under laboratory conditions on a small chamber and will be the basis for further studies.

## Figures and Tables

**Table 1:** Calculated values in critical input cross-section.

Mach Number	Output Velocity/Critical Velocity	Output Velocity/Input Velocity	Output Temperature/Input Temperature	Output Pressure/Input Pressure	Output Density/Input Density	Output Density/Critical Density
*M_v_*	*v_v_/v_kr_*	*v_v_/v_o_*	*T_v_/T_o_*	*p_v_/p_o_*	*ρ_v_/ρ_o_*	*ρ_v_/ρ_kr_*
2.6	1.8571	0.6521	0.4252	0.05	0.1179	0.3453

**Table 2:** Results of comparison of Ansys values with one-dimensional flow theory.

	Theoretical Value	Ansys Fluent Value
Mach number (-)	2.6	2.65
Density (kg·m^−3^)	0.5	0.49
Velocity (m·s^−1^)	598.6	600
Temperature (°C)	−146.6	−148

## Data Availability

The data presented in this study are available on request from the corresponding author.

## Nomenclature

**Quantity**	**Symbol**	**Unit**
Mach Number	*M*	-
Output Mach Number	*M_v_*	-
Input Velocity	*v_o_*	m·s^−1^
Output Velocity	*v_v_*	m·s^−1^
Critical Velocity	*v_kr_*	m·s^−1^
Input Pressure	*p_o_*	Pa
Output Pressure	*p_v_*	Pa
Input Density	*ρ_o_*	kg·m^−3^
Output Density	*ρ_v_*	kg·m^−3^
Location of The Mach Disk	*Z_m_*	mm
Computational Cross-Section	*A_kr_*	m^2^
Input Temperature	*T_o_*	K
Output Temperature	*T_v_*	K
Static Temperature	*T*	K
Stagnation Temperature	*T_stg_*	K
Poisson’s Ratio	ϰ	-
Universal Gas Constant	*R*	J·K^−1^·mol^−1^
Cone Angle	*c*	°
Deflection Angle	*a*	°
Ray Angle	*θ*	°
Shock Angle	*s*	°
